# Bucket-handle cervical tear at term following oxytocin-induced vaginal delivery: a case report

**DOI:** 10.1186/s13256-022-03269-y

**Published:** 2022-02-15

**Authors:** Abraham Fessehaye, Mariamawit Asfaw, Hika Hailu Kitila, Wondimu Gudu

**Affiliations:** Department of Obstetrics and Gynecology, Saint Paul’s Hospital millennium Medical College, Addis Ababa, Ethiopia

**Keywords:** Bucket handle, Cervical tear, Oxytocin induction

## Abstract

**Background:**

A bucket-handle cervical tear, a laceration of the anterior or the posterior lip of the cervix so that it hangs like the handle of a bucket, is the rarest type of cervical laceration. Our case represents such a serious cervical laceration.

**Case summary:**

A 28 year-old para 2 (both vaginal deliveries) Ethiopian mother presented at term with prolonged rupture of membrane. After 8 hours of oxytocin induction, a bucket-handle cervical detachment was detected at the time of her vaginal delivery, up on digital pelvic examination. Delivery of the baby was noted to be through the cervical tear, not the cervical opening. Cervix was amputated at the level of the cervical tear, 4 days after a failed initial repair surgery.

**Conclusion:**

When bucket-handle cervical tear is encountered, a thorough clinical evaluation of the viability of the cervical tissue and feasibility of a repair procedure should be made during the first surgery. If the cervical detachment is near total, as in our case, we recommend amputation of the hanging cervical tissue from the outset.

## Background

Cervical tears are a known cause of postpartum hemorrhage, and are associated with maternal morbidity and mortality if not identified early and managed timely [1]. Reported cervical injuries during labor include lateral cervical tears, bucket-handle tears, and annular detachment of the cervix [2]. Cervical trauma, or damage to the integrity of the cervical stroma, has been commonly cited as a contributing factor for cervical insufficiency and preterm birth [3]. A bucket-handle tear, a laceration of the anterior or the posterior lip of the cervix so that it hangs like the handle of a bucket, is the rarest type of cervical laceration [4]. We report a case of such a type of cervical laceration, which occurred during oxytocin-induced labor and delivery.

## Case presentation

A 28 year-old para 2 (both vaginal deliveries) Ethiopian mother delivered vaginally at term after 8 hours of induction of labor with oxytocin. The oxytocin dose was a high-dose oxytocin regimen. The indication for induction was prolonged term premature rupture of the membranes (PROM) and the outcome was an alive 2900 grams female neonate with an Apgar score of 7/10 and 8/10 in the 1st and 5th minutes, respectively. No prostaglandin was used for cervical ripening. Throughout the 8 hours of induction, she was in first stage of labor—the cervix was assessed as 3 cm and 80% effaced (pelvic examinations were done at 4 hour intervals). She had adequate uterine contractions—4–5 contraction that lasted 35–45 seconds. She was not on any form of pain medication (anesthesia). There was no cardiotocography (CTG) and labor was followed with intermittent fetal auscultation and manual monitoring of uterine contractions. Thirty minutes prior to delivery, her cervix was assessed and it was still at 3 cm dilation with no change in effacement. Then, after 30 minutes she was noted to have an urge to push with eminent delivery and presenting fetal was at station +3. There was no history of suggestive symptoms or clinical evidence of any cervical lesion, consequent fibrosis, or stenosis. The speculum and digital examinations during the current pregnancy did not indicate any pathology. Her past medical, surgical, and psychosocial history was unremarkable.

**Fig. 1:**
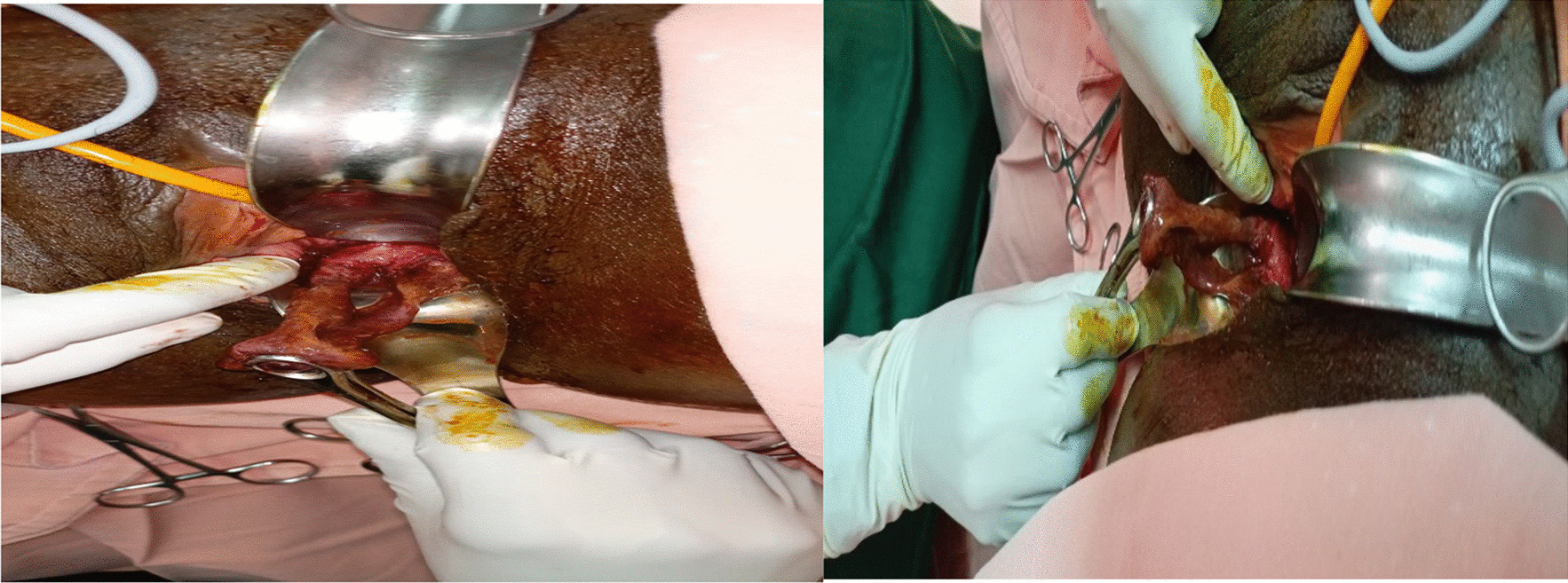
A bucket-handle cervical tear in our case

Immediately after her vaginal delivery, a bucket-handle cervical detachment was detected by the attending care provider upon digital pelvic examination, while the cervical dilatation remained at 3 cm. Delivery of the baby was noted to be through the cervical tear, not the cervical opening. She was taken to the operating theater and her cervix was explored under general anesthetic. The finding was a near-complete detachment of the cervix (only a small anterior part of the cervix was intact) at the level of 1 cm distal to the cervicovaginal junction (Fig. 1). There was no active vaginal bleeding and it was successfully repaired in anatomic position with a Vicryl 2-0 running technique (the circumferential detachment of the cervix was repaired with end-to-end anastomosis).

However, the repair failed 4 days later. A second operation was required to amputate  the hanging anterior part of the cervix and secure hemostasis. The indication for the amputation was a necrotized distal cervical tissue with loose attachment. She was put on intravenous antibiotics and was discharged after 3 days in good condition. She was counseled on having a transabdominal cervical cerclage in her future pregnancies, as the level of amputation was high, very close to the lower uterine segment. Sterile vaginal examination which was done a week later during her follow-up visit revealed good cervical wound healing.

## Discussion

Cervical tears have been frequently reported with instrumental delivery, particularly when forceps are engaged. However, large tears that mimic a full dilatation and lead to fetus delivery are very rare [4]. A bucket-handle cervical tear may be secondary to an unyielding cervix due to fibrosis or stenosis, leading to pressure being transferred either to the anterior or posterior lip of the cervix. An added factor may be hypertonus caused by prostaglandins (PR) [5]. In our patient, there was no history of suggestive symptoms or clinical evidence of any cervical lesion, consequent fibrosis, stenosis, or prostaglandin use.

Unlike in the current literature, based on few case reports, where the occurrence of such a cervical tear is ascribed to misoprostol (prostaglandin) use, in our case apart from oxytocin induction of labor there was no any prostaglandin administration. Djokovic *et al*. reported a spontaneous delivery through a cervical tear provoked by prostaglandin-induced uterine contractions in a G2P0 woman with a history of cervical dilatation and uterine curettage [6]. Singhal et al reported the first two cases of bucket-handle cervical tears in a second trimester unscarred uterus after misoprostol use [7], and Abubeker *et al*. reported a third similar case in 2020 [8].

During the serial digital examinations of the cervical response during induction of labor, particular attention should be paid to the characteristics and modification of the external os. Upon completion of cervical effacement and in the presence of significant uterine contractility, repetitive findings of a rigid external os should be considered as an alarm signal [6]. Our case was in the latent phase of labor throughout the induction of labor. The sudden discovery of an imminent vaginal delivery within 30 minutes—a dramatic jump in the course of labor from latent first stage to an eminent delivery within a short time interval demonstrates the reality of this alarm signal. The finding of the same cervical dilation immediately after delivery and the fact that the attending physician actually determined that delivery of the fetus occurred through the large cervical tear, not the cervical os, substantiates the importance of not missing a timely response to this warning sign.

Regardless of etiology, this complication could be prevented by performing a cesarean section upon recognition of the pathologic significance of external os rigidity (9). However; if it occurs, a meticulous assessment of the degree of the cervical tear should be made so that the feasibility of repair of the tear can determined during the first surgery. This could avoid a subsequent complication and a second operation, as was encountered in our case.

## Conclusion

Large cervical tears, such as bucket-handle tears, which mimic a full dilatation and lead to fetal delivery are very rare. When it is  is encountered, a thorough clinical evaluation of the viability of the cervical tissue and feasibility of a repair procedure should be made during the first surgery. If the cervical detachment is near total, as in our case, we recommend amputation of the hanging cervical tissue from the outset.

## Data Availability

All supporting documents are submitted along with the case report.
